# Sex-Specific Associations between Serum Ferritin and Osteosarcopenic Obesity in Adults Aged over 50 Years

**DOI:** 10.3390/nu14194023

**Published:** 2022-09-28

**Authors:** Sung-Joon Chung, Han Sol Lim, Mi-Yeon Lee, Yong-Taek Lee, Kyung Jae Yoon, Chul-Hyun Park

**Affiliations:** 1 Department of Physical and Rehabilitation Medicine, Kyunghee University Hospital at Gangdong, 892 Dongnam-ro, Gangdong-gu, Seoul 05278, Korea; 2 Department of Medicine, Graduate School, Kyunghee University, Seoul 02447, Korea; 3 Department of Physical and Rehabilitation Medicine, Kangbuk Samsung Hospital, Sungkyunkwan University School of Medicine, 29 Saemunan-ro, Jongno-gu, Seoul 03181, Korea; 4 Division of Biostatistics, Department of R&D Management, Kangbuk Samsung Hospital, Sungkyunkwan University School of Medicine, 29 Saemunan-ro, Jongno-gu, Seoul 03181, Korea; 5 Department of Health Sciences and Technology, SAIHST, Sungkyunkwan University, Seoul 06355, Korea

**Keywords:** ferritin, osteoporosis, sarcopenia, obesity, osteosarcopenic obesity, elderly

## Abstract

We investigated the sex-specific association between ferritin and adverse body composition in adults aged over 50 years in a population-based cohort. A total of 25,546 participants (16,912 women; 8634 men) were stratified into three groups by the tertiles of ferritin. The number of adverse body compositions was categorized as 0 (without osteopenia/osteoporosis, low muscle mass, or obesity), 1 (having one of the components), 2 (two), and 3 (all three; osteosarcopenic obesity). As ferritin tertile increased, the prevalence of one, two, or three simultaneous adverse body compositions increased, significant only in women (*p* < 0.0001), not in men (*p* = 0.125). Among women, the prevalence of osteosarcopenic obesity gradually increased from 1.7% in the lowest, to 2.2% in the middle, and 2.5% in the highest tertile. Using multivariate-adjusted analysis, women in the higher tertile had an increased likelihood of having multiple adverse body compositions compared with those in the lowest tertile. Women in the highest tertile had a 1.52 times increased risk of osteosarcopenic obesity than those in the lowest tertile. A high ferritin level was associated with an increased risk of having multiple adverse body compositions, especially for osteosarcopenic obesity in women aged >50 years, suggesting its potential use for detecting osteosarcopenic obesity.

## 1. Introduction

Osteosarcopenic obesity (OSO), a newly identified syndrome, is characterized by the simultaneous decline in bone (osteopenia or osteoporosis) and in muscle mass (sarcopenia), and excess adipose tissue (obesity) [1]. Bone and muscle mass decreases with aging, while body fat accumulates, leading to frailty, reduced functionality, and systemic metabolic dysfunction [2]. Osteoporosis/osteopenia, sarcopenia, and obesity are substantially interrelated with shared risk factors, such as aging, poor levels of physical activity, diabetes, and metabolic syndrome [2]. Recently, OSO has been associated with reduced functionality, low physical performance, low vitamin D levels, hypertension, and dyslipidemia [3,4,5,6,7]. It is well known that each OSO component is associated with many adverse health complications and chronic diseases. However, OSO, as a combined disease state, can lead to even more adverse health outcomes, and one component may cause another. Adverse outcomes include physical disability, impaired quality of life, fracture, insulin resistance, length of hospital stay, and mortality [2]. Furthermore, all independent OSO components are associated with significantly greater financial burden [8,9]. These adverse outcomes and healthcare costs seem to become worse when compared to individuals with only one adverse body composition. Therefore, the coexistence of these three tissue impairments, OSO, which can potentiate each other as a triple metabolic burden, has been given special attention.

Iron overload has been known as a potential health risk that can cause oxidative stress and cellular membrane damage by the generation of reactive oxygen species [10,11]. Ferritin is a highly sensitive biomarker of iron storage in the body [12]. The iron is stored in the form of ferritin and hemosiderin, mostly in the liver and in the spleen, the duodenum, marrow, skeletal muscle, and other anatomic areas [13]. Although low ferritin is typically used as a marker of iron deficiency, an increased ferritin level is highly associated with elevated C-reactive protein (CRP) levels and chronic inflammation. Recently, ferritin has been shown to act as a proliferative mediator of hepatocytes and is known to interact with numerous plasma proteins involved in the host response to sepsis. [14,15]. Therefore, ferritin is suggested to be an acute-phase reactant, which can potentially be elevated in an inflammatory condition [16]. A few studies have reported that high serum ferritin levels are closely related to metabolic syndrome, obesity, impaired muscle quality, and sarcopenia [17,18,19,20]. A population study demonstrated that ferritin is inversely associated with bone mineral density (BMD) in older women [21]. Iron accumulation is known to occur easily in the elderly population and can concurrently impair bone metabolism, muscle loss, and fat accumulation [22,23,24,25]. We hypothesized that serum ferritin can be served as a biomarker that could reflect osteosarcopenic obesity, a combination of various body composition abnormalities.

Therefore, the present study was to investigate the association of serum ferritin levels with combined abnormal body compositions (osteopenia/osteoporosis, sarcopenia, and obesity) in women and men aged >50 years.

## 2. Materials and Methods

### 2.1. Study Participants

This was a two-center, cross-sectional study from a cohort of the Kangbuk Samsung Health Study (KSHS). The study included participants through a medical health screening program at the Kangbuk Samsung Hospital Healthcare Centers and Sungkyunkwan University College of Medicine in Seoul and Suwon, Republic of Korea.

**Figure 1:**
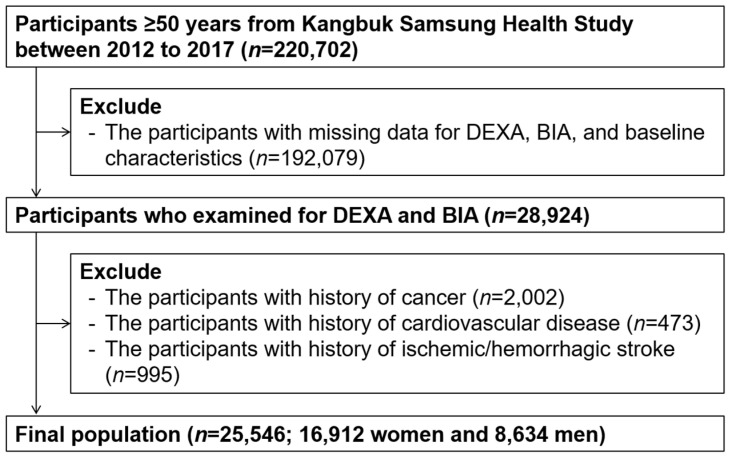
Selection of study participants.

The study participants were adults aged over 50 years who underwent both bioelectrical impedance analysis (BIA) and dual energy X-ray absorptiometry (DXA) from 1 January 2012 to 31 December 2017 (*n* = 220,702). Participants who met the exclusion criteria were as follows: (1) missing data for baseline characteristics (*n* = 192,079), (2) history of malignancy (*n* = 2,002), (3) history of cardiovascular disease (*n* = 995), and (4) history of ischemic or hemorrhagic stroke (*n* = 47). Some participants met one or more exclusion criteria, leaving 25,546 participants included in the final analysis (Figure 1). The study protocol was performed in accordance with the Declaration of Helsinki and was approved by the Institutional Review Board (IRB) of Kangbuk Samsung Hospital (IRB no. KBSMC 2021-12-047). The requirement for prior consent was waived by the IRB as we accessed anonymized datasets collected regularly as part of the health screening exam.

### 2.2. Measurements

Study data included medical history (hypertension, diabetes mellitus, and dyslipidemia), health-related behavior variables (heavy drinking, current smoker, and regular physical activity), anthropometric measurements, and laboratory measurements. Medical history and health-related behaviors were collected by physicians using standardized questionnaires. Those who consumed more than 20 g of alcohol a day were classified as heavy drinkers. Smoking status was categorized as never, former, or current smokers. Physical activity was evaluated by using the International Physical Activity Questionnaire—Short Form. Participants who performed moderate exercise ≥5 times per week for >30 min per session or vigorous exercise ≥3 times per week for >20 min per session were categorized into the regular physical activity group [26].

Trained nurses performed anthropometric and laboratory measurements. Each individual’s weight and height were measured twice and averaged. Body mass index (BMI) was calculated as the weight in kilograms divided by the square of height in meters (kg/m^2^).

Blood samples for laboratory measurements were collected after an overnight fast of >12 h. Serum ferritin levels were measured using electrochemiluminescence immunoassay (Cobas 8000 e602; Roche Diagnostics). Serum iron and total iron-binding capacity (TIBC) levels were measured using an automatic chemistry analyzer (Cobas 8000 c702; Roche Diagnostics, Tokyo, Japan) and colorimetric assay. Normal range of serum ferritin level was 40–200 ng/mL. High ferritin level was defined as over 200 ng/mL, and low ferritin level as below 40 ng/mL. Normal range of serum iron was 60–150 ng/mL. High serum iron level was defined as over 150 ng/mL and low iron level as below 60 ng/mL [27,28].

Total cholesterol and triglyceride levels were measured by the enzymatic colorimetric method. High-density lipoprotein cholesterol (HDL) and low-density lipoprotein cholesterol (LDL) measurement were performed by the elective inhibition method and the homogeneous enzymatic colorimetric test, respectively. For the measurement of glycated hemoglobin (Hba1c), an immunoturbidimetric assay (Cobra Integra 800 automatic analyzer, Roche Diagnostics, Basel, Switzerland) was used. Aspartate transaminase (AST) and alanine aminotransferase (ALT) levels were measured by the Bayer Reagent Packs on an automated chemistry analyzer (Advia 1650 autoanalyzer, Bayer Diagnostics, Leverkusen, Germany). Serum creatinine level was measured using the alkaline picrate (Jaffe) method. For the measurement of serum albumin, the Bromocresol green dye-binding method, using Bayer reagent packs on an automated chemistry analyzer (Advia 1650 Autoanalyzer, Bayer Diagnostics, Leverkusen, Germany) was used.

To find out the association and trend with the adverse body composition (e.g., OSO), serum ferritin concentrations were divided into an ordered distribution based on methods of previous studies [29,30,31,32]. Hence, we applied natural log-transformed (log) ferritin values, which are required for the comparison of ferritin levels, as a continuous variable.

### 2.3. Determination of Adverse Body Composition and Osteosarcopenic Obesity

Bone mineral densities were measured using DXA for the lumbar spine (L2–L4) and total hip (Lunar Prodigy; GE, Madison, WI, USA) [33,34,35,36]. Calibration was performed using a machine-specific phantom each day prior to examination. Osteopenia/osteoporosis was defined by the World Health Organization (WHO) criteria: osteopenia (T-score from −2.4 to 1.0) and osteoporosis (T-score ≤ −2.5) [37].

Appendicular skeletal muscle mass (ASM) (kg) and percentage fat mass (%) were estimated using BIA using eight-point tactile electrodes (InBody 720, Biospace, South Korea). Before the examination, the BIA was calibrated every morning and validated for accuracy and reproducibility in assessing body composition. Appendicular skeletal muscle mass is the summation of the muscle masses of the arms and legs. Skeletal muscle mass index (SMI) was calculated by the following formula: SMI (kg/m^2^) = ASM (kg)/height (m^2^). Low muscle mass was defined as ASM more than 1 standard definition (SD) below the sex-specific mean of a young reference group [5,38,39]. Obesity was defined as percent body fat (%) using cutoff levels of 35% for women and 25% for men based on a previous study [40,41].

OSO is defined as the co-presence of osteopenia/osteoporosis, low muscle mass, and obesity, which were determined based on the above definitions [5]. The number of adverse body compositions was categorized as 0 (without osteopenia/osteoporosis, low muscle mass, or obesity), 1 (having one of the adverse body components), 2 (having two of the components), and 3 (OSO, osteosarcopenic obesity; having all three components).

### 2.4. Statistical Analysis

Baseline variables for the groups were compared using the X^2^ test for categorical variables and the Student t-test for continuous variables. The study subjects were grouped based on the tertile (T) distribution of serum ferritin as T1 (lowest tertile), T2 (middle), and T3 (highest). The prevalence of adverse body composition in each group classified by tertiles of serum ferritin was compared using the X^2^ test and post hoc analysis corrected by the Bonferroni method. The analysis was repeated for ferritin groups classified by cutoff levels as low ferritin, normal ferritin, high ferritin groups with prevalence of multiple abnormal body composition. Means (±SD) of log-transformed ferritin values according to the number of abnormal body compositions were compared using the one-way ANOVA and post hoc Bonferroni analysis.

The multivariate logistic regression analysis was used for the odds ratio (OR) and 95% confidence interval (CI) of unfavorable body compositions with tertiles of ferritin concentration after adjustments for age, hypertension, fasting glucose, heavy drinker, smoking status, LDL, ALT, serum creatinine, and regular physical activity. The multivariate logistic regression analysis was repeated for association of adverse body composition with ferritin group according to cutoff value. The level of statistical significance was set at a two-tailed *p*-value < 0.05. All the analyses were performed by SPSS version 26.0 (IBM Co., New York, NY, USA).

## 3. Results

### 3.1. Baseline Characteristics of the Study Population

A total of 25,546 participants aged over 50 years were included, consisting of 16,912 (66.2%) women and 8634 (33.8%) men. The mean age was 58.3 (SD, 6.4) in women and 59.6 (6.8) years in men. The mean serum ferritin was 111.38 (SD, 76.55) and 228.19 (SD, 154.56), in women and men, respectively (Table 1). The serum ferritin levels were categorized as the following tertiles (T); for women: T1 (<74.50 ng/mL), T2 (≥74.5 and <125.0), and T3 (≥125.00) and for men: T1 (<153.5 ng/mL), T2 (≥153.5 and <248.0), and T3 (≥248.0) (Supplementary Table S1). The distribution of serum ferritin levels is positively skewed. Baseline characteristics of women based on tertiles of serum ferritin level are described in Supplementary Tables S1 and S2.

### 3.2. Relationship of Serum Ferritin with Adverse Body Composition Including Osteosarcopenic Obesity

Among women, for the number of abnormal body compositions, 31.6% had none, 45.2% had one, 21.1% had two, and 2.1% had all three (=OSO), respectively. In men, it was 34.3%, 44.4%, 18.2%, and 3.1%, respectively. The prevalence of one, two, or three simultaneous adverse body compositions increased as the tertile of serum ferritin increased, which was significant only in women, not in men (*p* for trend < 0.0001 in women; *p* for trend = 0.125 in men) (Table 2). Among women, the prevalence of OSO gradually increased from 1.7% in the lowest tertile, to 2.2% in the middle tertile, and 2.5% in the highest tertile of ferritin (*p* for trend < 0.0001). Furthermore, the analyses were repeated for ferritin groups by ferritin cutoff levels as low ferritin, normal ferritin, and high ferritin groups with the prevalence of multiple adverse body compositions. We found that the prevalence of one, two, or three simultaneous adverse body compositions increased as the ferritin group according to cutoff level became higher, with the prevalence of OSO highest in the high-level serum ferritin group. This positive trend was only significant in women, not in men (*p* for trend < 0.0001 in women; *p* for trend = 0.419 in men) (Table S3).

**Figure 2:**
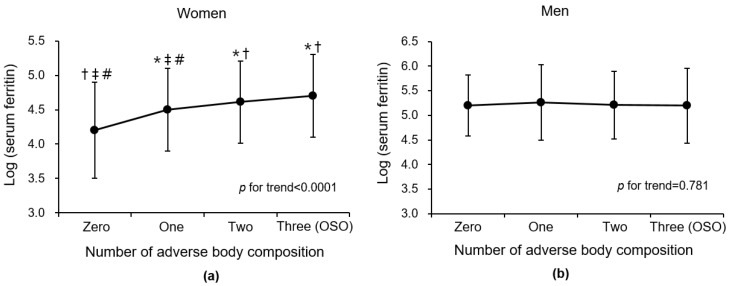
Comparison of mean (±SD) log-transformed ferritin according to number of adverse body compositions (osteoporosis/osteopenia, muscle mass loss, and obesity) in (**a**) women and (**b**) men. Abbreviations: OSO, osteosarcopenic obesity; SD, standard deviation. * *p* < 0.05 versus zero group in post hoc analysis. † *p* < 0.05 versus one group in post hoc analysis. ‡ *p* < 0.05 versus two group in post hoc analysis. # *p* < 0.05 versus three group in post hoc analysis.

When serum ferritin was introduced as a continuous variable, the mean of log (ferritin) was highest in participants with three (OSO) adverse body compositions, followed by that of participants with two, followed by one, and zero among women (*p* for trend < 0.0001) (Figure 2). In men, there was no significant linear trend between the log (ferritin) and number of adverse body compositions (*p* for trend = 0.781). These gender differences are consistent with the results of analysis by dividing serum ferritin into tertiles.

Table 3 presents the risks of having unfavorable body compositions with tertiles of ferritin values. In a multivariate logistic analysis after adjustments for age, hypertension, fasting glucose, heavy drinker, smoking status, LDL, ALT, serum creatinine, and regular physical activity, women in the higher tertile of ferritin values, compared to those in the tertile with the lowest, had a significantly increased likelihood of numerous abnormal body compositions (all *p* for trend < 0.0001 for those with one, two, or three adverse composition compared to zero). For women, those in the highest tertile of ferritin had a 1.52 times increased risk of having OSO compared to those in the lowest tertile group (adjusted OR 1.52; 95% CI, 1.07–2.14). Those in the middle tertile (T2) had a 1.46 times increased risk of having OSO compared to those in the lowest tertile group (adjusted OR 1.46; 95% CI 1.03–2.06). For men, participants with higher tertiles of ferritin levels, compared with the lowest tertile, had no significant trend for having multiple adverse components (*p* for trend = 0.757). Furthermore, there was no association of having OSO with higher tertiles of ferritin in men.

The analysis was repeated using ferritin groups by the cutoff values as low, normal, and high ferritin groups; women in the higher serum ferritin level group had a higher risk of numerous adverse body compositions, and it was consistent after adjustment for covariates (Table S4). In women, those in the high ferritin group had a 2.35 times increased risk of having OSO compared to those in the low ferritin group (adjusted OR 2.35; 95% CI, 1.22–4.51). Those in the normal ferritin group had a 2.00 times increased risk of having OSO compared to those in the lowest tertile group (adjusted OR 2.00; 95% CI 1.22–4.51). In men, there was no significant association of having OSO with the high ferritin level group.

## 4. Discussion

Serum ferritin concentrations were associated with combined adverse body compositions in elderly women aged >50 years, but not in elderly men. The prevalence of combined adverse body compositions (one, two, or three) increased as ferritin levels increased. After controlling for potential covariates, the risk of OSO was higher in women with the highest tertile than in those with the lowest tertile. To the best of our knowledge, this is the first population study to demonstrate sex-specific associations between serum ferritin levels and OSO in adults aged >50 years.

Iron is an essential inorganic substance involved in human physiology. However, excess iron could result in the formation of free radicals, which may cause oxidative damage in diverse tissues [10]. In thalassemia, it is well known that iron overload can damage myocardial cells, causing cardiomyopathy. According to the possible theory of mechanisms, iron overload causes an excess production of reactive oxygen species in the mitochondria, which could alter the physiologic activity of the osteoclast and osteoblast, leading to an imbalance of bone homeostasis. [40]. This oxidative stress may increase susceptibility to apoptosis during the development of sarcopenia [18,29] and lead to inflamed adipose tissue during the aggravation of obesity [41]. The mechanism of OSO is thought to begin with mesenchymal stem cell lineage deregulation [1], and a recent study reported that the osteogenic commitment and differentiation of mesenchymal stem cells is hindered through the induction of ferritin by excess iron. [42].

Animal studies have reported various adverse effects of iron overload on bone, muscle, and fat. One study reported that iron overload in a murine model causes thinning of the trabeculae and cortex and changes in the material properties of the bone [43]. A study in mice that administered an intraperitoneal injection of iron dextran showed significant increases in muscular iron and ferritin and reductions in body weight, skeletal muscle, and amounts of fast-twitch muscle fibers [44]. It is described that a dietary iron supplement in combination with a high-fat diet significantly increases adiposity in Wistar rats [45].

Extra iron in the body can be stored in the iron storage protein ferritin, which is present in cells and in the circulatory system. Various studies have reported the association of serum ferritin levels with adverse body compositions. A recent review of the relationship between serum ferritin levels and metabolic syndrome reported a significant positive association [46]. A study on a sample of Korean older women represented nationally reported that higher serum ferritin levels were associated with the prevalence of sarcopenia [29]. Moreover, it has been reported that serum ferritin concentrations are negatively associated with bone mineral density values in Korean women ≥45 years of age [21]. The above studies endorsed our result that increased serum ferritin levels are associated with the prevalence of OSO, which combined osteoporosis, sarcopenia, and obesity.

In our study, the relationship between serum ferritin and OSO was more pronounced in older women than in older men. Furthermore, women had a positive trend between serum ferritin and the number of adverse body compositions, which was not seen in men. Similar to our study, a few studies on the association between serum ferritin and metabolic disorders, including obesity, sarcopenia, and osteopenia, have shown a significant association in women [21,29,47]. There are some possible explanations for gender-specific differences. First, adipose tissue-derived hormones, such as leptin, exist at higher levels in women than in men [48,49]. Second, the distribution and metabolism of body fat and skeletal musculature are quite different between men and women, which also influences their relationship with ferritin levels [50,51]. Thus, the differences in the hormonal and metabolic effects of muscle and adiposity may affect sex differences in ferritin and OSO. Furthermore, the findings of our study are in line with those of a previous report, which demonstrated a stronger association between high-sensitivity CRP and sarcopenic obesity in asymptomatic women than in men [52]. Nevertheless, various hypotheses have been suggested, but the exact mechanism has not yet been clarified and needs to be further studied.

The knowledge of OSO is still limited and the prevalence is not known exactly. In a Korean cohort study, the prevalence of OSO was reported as 3.1% for men and 5.4% for women [53], but another study reported this as 13.5% for men and 25% for women [5]. In a recent Chinese study, it was reported as 15.7% for men and 7.9% for women [6]. In our study, the prevalence was 3.1% in men and 2.1% in women. These differences between the studies are thought to be due to differences in the diagnostic criteria and measurement methods of OSO components as well as differences in the study populations. Considering the growing interest and accurate understanding of OSO, it is necessary to clarify the diagnostic criteria of OSO through additional research.

Our study has a few limitations. First, the cross-sectional study design makes it difficult to identify a causation between serum ferritin and OSO. Second, our results were derived from relatively healthy older adults, who participated in a health checkup program, and may not be representative of the whole Korean population. As a result, the prevalence of OSO in this study may be lower than that found in previous studies [38], and it may not have accurately reflected the actual prevalence. Third, we used the fat mass cutoff value of previous studies as a criterion for obesity [38]; however, there is still debate regarding the assessment of obesity, and it is thought that ethnic differences could not be reflected. Fourth, there was a disproportionateness between the number of women and men, with more participants being women. Further study between similar male and female participants is needed. Fifth, other baseline variables were not considered in this study such as nutrient status and the eating disorder anorexia nervosa, which could affect muscle and fat metabolism. Further study including these variables is needed. Sixth, individuals with chronic liver disease or autoimmune disease, such as SLE and RA, can show higher serum ferritin levels than those without. In this study, however, we could not exclude this disease due to the study design and there being no information. Further studies that exclude autoimmune disease and chronic liver disease or consider them as a contributing factor are needed. Lastly, conditions of women such as low estrogen levels or menopause state can be associated with bone and muscle loss. Further study should include other contributing factors, such as leptin, estrogen, and menopause state.

## 5. Conclusions

We found sex-specific differences in the relationship between ferritin and OSO, which showed that elevated ferritin levels are strongly associated with having OSO only in women aged over 50 years. Our findings suggest that serum ferritin may potentially be used as a biomarker for combined adverse body compositions, especially for OSO in women. For clinicians, women with an increased ferritin level should be evaluated for abnormal body compositions. Further research is needed to identify whether high serum ferritin level is a strong risk factor for OSO occurrence with a population-based longitudinal study and an optimal threshold value of serum ferritin level associated with an increased risk of OSO occurrence.

## Figures and Tables

**Table 1:** Baseline characteristics of study population in women and men.

Characteristics	Total	Women	Men	*p*-Value ^a^
Number of subjects (*n*)	25,546	16,912	8634	
Age (years)	58.74 (6.58)	58.28 (6.44)	59.64 (6.76)	<0.0001
Height (cm)	160.79 (7.91)	156.63 (5.25)	168.94 (5.54)	<0.0001
Weight (kg)	61.16 (10.15)	56.98 (7.87)	69.35 (9.07)	<0.0001
BMI (kg/m^2^)	23.58 (2.98)	23.23 (3.04)	24.27 (2.73)	<0.0001
Fat mass (kg)	18.24 (5.51)	18.98 (5.52)	16.78 (5.19)	<0.0001
Percent body fat (%)	29.75 (7.05)	32.78 (5.87)	23.83 (5.16)	<0.0001
ASM (kg)	17.60 (4.64)	15.19 (1.97)	22.33 (4.74)	<0.0001
SMI ^b^ (kg/m^2^)	6.72 (1.27)	6.17 (0.58)	7.80 (1.53)	<0.0001
Current smoker (%)	10.4 (*n* = 2296)	1.7 (*n* = 236)	25.9 (*n* = 2060)	<0.0001
Heavy drinking ^c^ (%)	16.5 (*n* = 3509)	3.2 (*n* = 432)	39.5 (*n* = 3077)	<0.0001
Regular physical activity ^d^ (%)	23.4 (*n* = 5968)	21.8 (*n* = 3692)	26.4 (*n* = 2276)	<0.0001
Comorbidities				
Hypertension (%)	24.8 (*n* = 6343)	21.1 (*n* = 3561)	32.2 (*n* = 2782)	<0.0001
Diabetes (%)	8.6 (*n* = 2207)	6.4 (*n* = 1076)	13.1 (*n* = 1131)	<0.0001
Dyslipidemia (%)	29.9 (*n* = 7631)	29.7 (*n* = 5030)	30.1 (*n* = 2601)	0.527
Laboratory findings				
Ferritin (ng/mL)	150.86 (122.50)	111.38 (76.55)	228.19 (154.56)	<0.0001
Low (<40) (%)	8.8 (*n* = 2214)	12.0 (*n* = 2002)	2.5 (*n* = 212)	
Normal (40–200) (%)	68.4 (*n* = 17,273)	78.2 (*n* = 13,059)	49.2 (*n* = 4214)	<0.0001
High (200≤) (%)	22.9 (*n* = 5774)	9.8 (*n* = 1633)	48.3 (*n* = 4141)	
Iron (μg/dL)	111.61 (37.48)	104.25 (33.92)	126.02 (39.85)	<0.0001
Low (<60) (%)	6.3 (*n* = 844)	8.0 (*n* = 701)	3.2 (*n* = 143)	
Normal (60–150) (%)	80.6 (*n* = 10,726)	83.9 (*n* = 7385)	74.1 (*n* = 3341)	0.258
High (150≤) (%)	13.1 (*n* = 1739)	8.1 (*n* = 713)	22.7 (*n* = 1026)	
TIBC (μg/dL)	306.18 (41.55)	307.96 (42.08)	302.72 (40.26)	<0.0001
Total cholesterol (mg/dL)	200.90 (37.88)	205.18 (37.38)	287.98 (36.63)	<0.0001
LDL (mg/dL)	132.01 (35.59)	134.36 (35.72)	127.39 (34.86)	<0.0001
HDL (mg/dL)	60.48 (16.44)	63.84 (16.34)	53.90 (14.53)	<0.0001
Triglycerides (mg/dL)	111.12 (66.95)	103.98 (58.82)	125.11 (78.67)	<0.0001
Fasting glucose (mg/dL)	99.24 (17.18)	97.40 (15.59)	102.85 (19.44)	<0.0001
HbA1c (%)	5.78 (0.64)	5.75 (0.58)	5.85 (0.73)	<0.0001
Creatinine (mg/dL)	0.78 (0.22)	0.69 (0.15)	0.95 (0.23)	<0.0001
Albumin (g/dL)	4.60 (0.25)	4.58 (0.25)	4.64 (0.25)	<0.0001
AST (IU/L)	25.20 (12.40)	24.21 (10.75)	27.15 (14.93)	<0.0001
ALT (IU/L)	23.73 (15.09)	21.59 (13.42)	27.94 (17.15)	<0.0001

Abbreviations: ALT, alanine aminotransferase; ASM, appendicular skeletal muscle mass; AST, aspartate aminotransferase; HDL, high-density lipoprotein cholesterol; LDL, low-density lipoprotein cholesterol; SMI, skeletal muscle mass index. ^a^ Using Chi-square test for categorical variable or Student t-test for continuous variable. Values are presented as mean (standard deviation) or percentage (number of participants). ^b^ SMI = ASM/height (m^2^). ^c^ ≥ 20 g/day. ^d^ Moderate physical activity ≥ 5/week or vigorous physical activity ≥ 3/week.

**Table 2:** Prevalence of multiple adverse body compositions according to tertiles of serum ferritin in women and men.

Number of Adverse Body Compositions	Tertiles (T) of Serum Ferritin (ng/mL)			*p* for Trend
Women (*n* = 16,912)	T1 (*n* = 5556) (<74.50)	T2 (*n* = 5568) (≥74.5, <125.0)	T3 (*n* = 5570) (≥125.0)	
0 (%)	39.3 (2182)	29.7 (1652)	25.8 (1437)	<0.0001
1 (%)	42.0 (2336)	45.5 (2531)	48.2 (2684)	
2 (%)	17.0 (943)	22.7 (1263)	23.5 (1309)	
3 (=OSO) (%)	1.7 (95)	2.2 (122)	2.5 (140)	
Men (*n* = 8634)	T1 (*n* = 2862)	T2 (*n* = 2855)	T3 (*n* = 2850)	
	(<153.5)	(≥153.5, <248.0)	(≥248.0)	
0 (%)	35.5 (1015)	36.8 (1050)	30.7 (876)	0.125
1 (%)	42.5 (1216)	42.1 (1202)	48.5 (1382)	
2 (%)	18.6 (531)	18.5 (528)	17.5 (498)	
3 (=OSO) (%)	3.5 (100)	2.6 (75)	3.3 (94)	

Values are presented as percentage (number of participants). Abbreviations: OSO, osteosarcopenic obesity.

**Table 3:** Multivariate logistic regression analysis for association of ferritin tertiles (T) with multiple adverse body compositions in women and men.

Adverse Body Composition ^a^	Crude		Adjusted OR ^b^	
	OR (95% CI)	*p* for Trend	OR (95% CI)	*p* for Trend
Women (*n* = 16,912)				
1 (vs. 0)		<0.0001		<0.0001
T1 (lowest)	1 (ref.)		1 (ref.)	
T2 (middle)	1.43 (1.31–1.55)		1.20 (1.08–1.34)	
T3 (highest)	1.74 (1.60–1.90)		1.33 (1.20–1.49)	
2 (vs. 0)		<0.0001		<0.0001
T1	1 (ref.)		1 (ref.)	
T2	1.76 (1.59–1.96)		1.36 (1.19–1.56)	
T3	2.10 (1.89–2.34)		1.43 (1.24–1.64)	
3 (=OSO) (vs. 0)		<0.0001		0.014
T1	1 (ref.)		1 (ref.)	
T2	1.69 (1.28–2.23)		1.46 (1.03–2.06)	
T3	2.23 (1.71–2.92)		1.52 (1.07–2.14)	
Men (*n* = 8634)				
1 (vs. 0)		0.147		0.411
T1	1 (ref.)		1 (ref.)	
T2	0.95 (0.85–1.07)		0.96 (0.84–1.10)	
T3	1.31 (1.16–1.48)		1.23 (1.07–1.41)	
2 (vs. 0)		0.946		0.285
T1	1 (ref.)		1 (ref.)	
T2	0.96 (0.82–1.11)		1.04 (0.88–1.23)	
T3	1.08 (0.93–1.26)		1.17 (0.98–1.40)	
3 (=OSO) (vs. 0)		0.230		0.757
T1	1 (ref.)		1 (ref.)	
T2	0.72 (0.53–0.99)		0.91 (0.63–1.30)	
T3	1.08 (0.81–1.46)		1.33 (0.94–1.90)	

Abbreviations: ALT: alanine aminotransferase; CI: confidence interval; LDL: low-density lipoprotein cholesterol; OR: odds ratio; OSO: osteosarcopenic obesity. ^a^ Number of osteoporosis/osteopenia, muscle mass loss, and obesity. ^b^ Adjusted for age, hypertension, fasting glucose, heavy drinker, smoking status, LDL, ALT, serum creatinine, and regular physical activity. Serum ferritin tertile (T) levels for women: T1 (<74.50), T2 (≥74.5, <125.0), and T3 (≥125.0). Serum ferritin tertile (T) levels for men: T1 (<153.5), T2 (≥153.5, <248.0), and T3 (≥248.0).

## Data Availability

Data can be obtained from corresponding author upon reasonable request.

## Supplementary Materials

The following supporting information can be downloaded at: https://www.mdpi.com/article/10.3390/nu14194023/s1, Table S1: Baseline characteristics of women according to tertiles of serum ferritin level. Table S2: Baseline characteristics of men according to tertiles of serum ferritin level. Table S3: Prevalence of multiple adverse body compositions according to ferritin group according to cutoff value in women and men. Table S4: Multivariate logistic regression analysis for association of ferritin group according to cutoff value with multiple adverse body compositions in women and men.
