# Supplementary material for: Sex-Specific Associations between Serum Ferritin and Osteosarcopenic Obesity in Adults Aged over 50 Years
Source: Nutrients. 2022 Sep 28;14(19):4023. doi: 10.3390/nu14194023 (PMC9570979; doi:10.3390/nu14194023)
Supplement: Supplementary file 1 [file nutrients-14-04023-s001.zip › nutrients-1884767-supplementary.pdf]

Supplementary Table 1. Baseline characteristics of women according to tertiles of serum ferritin level.

Characteristics	Total	Tertiles (T) of serum ferritin			*p* for trend
		T1	T2	T3	
No. of subjects (n)	16,912	5,636	5,637	5,639	
Age (years)	58.28 (6.44)	58.85 (6.62)	58.68 (6.32)	59.31 (6.12)	<0.0001
Height (cm)	156.63 (5.25)	157.00 (5.28)	156.55 (5.20)	156.32 (5.25)	<0.0001
Weight (kg)	56.98 (7.87)	56.46(7.81)	56.60 (7.48)	57.89 (8.21)	<0.0001
BMI (kg/m^2)	23.23 (3.04)	22.91 (3.00)	23.09 (2.88)	23.69 (3.17)	<0.0001
Fat mass (kg)	18.98 (5.52)	18.31 (5.48)	18.78 (5.27)	19.84 (5.69)	<0.0001
Percent body fat (%)	32.78 (5.87)	31.90 (5.99)	32.70 (5.72)	33.74 (5.75)	<0.0001
ASM (kg)	15.19 (1.97)	15.26 (2.00)	15.09 (1.91)	15.21 (1.99)	0.158
SMI[a] (kg/m^2)	6.17 (0.58)	6.17 (0.58)	6.14 (0.55)	6.20 (0.60)	0.002
Current smoker (%)	1.7	1.4	1.6	2.1	0.010
Heavy drinking[b] (%)	3.2	2.6	3.0	4.0	<0.0001
Regular physical activity[c] (%)	21.8	22.5	22.5	20.5	0.011
Comorbidities					
Hypertension (%)	21.1	18.8	21.6	22.8	<0.0001
Diabetes (%)	6.4	7.2	6.0	5.9	0.003
Dyslipidemia (%)	29.7	26.1	30.1	33.0	<0.0001
Laboratory findings					
Ferritin (ng/mL)	111.38 (76.55)	45.68 (20.23)	98.40 (14.38)	190.01 (79.35)	<0.0001
Iron (ug/dL)	104.25 (33.92)	101.55 (38.69)	106.26 (30.39)	105.14 (31.57)	<0.0001
TIBC (ug/dL)	307.96 (42.08)	326.44 (47.17)	300.18 (34.14)	296.17 (36.43)	<0.0001
Total cholesterol (mg/dL)	205.18 (37.38)	202.76 (36.45)	205.23 (36.33)	207.55 (39.15)	<0.0001
LDL (mg/dL)	134.36 (35.72)	130.74 (34.42)	134.00 (34.71)	138.34 (37.55)	<0.0001
HDL (mg/dL)	63.84 (16.34)	65.23 (16.48)	64.46 (16.23)	61.83 (16.12)	<0.0001
Triglycerides (mg/dL)	103.98 (58.82)	99.78 (52.36)	101.32 (53.15)	110.84 (68.87)	<0.0001
Fasting glucose (mg/dL)	97.40 (15.59)	96.28 (14.06)	96.67 (14.47)	99.24 (17.80)	<0.0001
HbA1c (%)	5.75 (0.58)	5.72 (0.53)	5.73 (0.50)	5.81 (0.68)	<0.0001
Creatinine (mg/dL)	0.69 (0.15)	0.68 (0.11)	0.69 (0.11)	0.70 (0.20)	<0.0001
Albumin (g/dL)	4.58 (0.25)	4.53 (0.24)	4.58 (0.24)	4.63 (0.25)	<0.0001
AST (IU/L)	24.21 (10.75)	22.26 (7.86)	23.72 (8.69)	26.64 (14.11)	<0.0001
ALT (IU/L)	21.59 (13.42)	18.84 (9.16)	20.75 (10.89)	25.17 (17.80)	<0.0001

Values are presented as mean (standard deviation) or percentage.

[a] SMI=ASM/height(m^2).

[b] ≥20g/day.

[c] Moderate physical activity≥5/week or vigorous physical activity≥3/week

ALT: alanine aminotransferase; ASM: appendicular skeletal muscle mass; AST: aspartate aminotransferase; HDL: high density lipoprotein cholesterol; LDL: low density lipoprotein cholesterol; SMI: skeletal muscle mass index.

Serum ferritin tertile (T) levels for women: T1 (<74.50), T2 (≥74.5, <125.0), and T3 (≥125.00).

Supplementary Table 2. Baseline characteristics of men according to tertiles of serum ferritin level.

Characteristics	Total	Tertiles (T) of serum ferritin			*p* for trend
		T1	T2	T3	
No. of Subjects (n)	8,634	2,878	2,878	2,878	
Age (years)	59.64 (6.76)	60.66 (7.14)	59.39 (6.58)	58.88 (6.40)	<0.0001
Height (cm)	168.94 (5.54)	168.33 (5.54)	169.09 (5.52)	169.42 (5.51)	<0.0001
Weight (kg)	69.35 (9.07)	67.55 (8.77)	68.96 (8.73)	71.54 (9.23)	<0.0001
BMI (kg/m^2)	24.27 (2.72)	23.82 (2.72)	24.09 (2.63)	24.89 (2.72)	<0.0001
Fat mass (kg)	16.78 (5.19)	15.78 (5.10)	16.48 (5.01)	18.08 (5.18)	<0.0001
Percent body fat (%)	23.83 (5.16)	22.98 (5.36)	23.57 (4.99)	24.93 (4.91)	<0.0001
ASM (kg)	22.33 (4.74)	21.95 (2.66)	22.26 (2.64)	22.77 (7.28)	<0.0001
SMI[a] (kg/m^2)	7.80 (1.53)	7.72 (0.64)	7.76 (0.62)	7.91 (2.50)	<0.0001
Current smoker (%)	25.9	23.6	26.2	28.0	<0.0001
Heavy drinking[b] (%)	39.5	31.4	39.6	47.0	<0.0001
Regular physical activity[c] (%)	26.4	28.1	26.7	24.3	0.001
Comorbidities					
Hypertension (%)	32.2	32.4	29.9	34.3	0.121
Diabetes (%)	13.1	16.3	11.6	11.4	<0.0001
Dyslipidemia (%)	30.1	27.5	30.0	32.8	<0.0001
Laboratory findings					
Ferritin (ng/mL)	228.19 (154.56)	102.69 (36.24)	197.11 (26.84)	384.77 (168.54)	<0.0001
Iron (ug/dL)	126.02 (39.85)	124.53 (40.07)	126.17 (38.45)	127.15 (40.90)	0.191
TIBC (ug/dL)	302.72 (40.26)	312.22 (43.68)	299.73 (37.15)	197.32 (38.52)	<0.0001
Total cholesterol (mg/dL)	287.98 (36.63)	187.89 (36.63)	194.08 (36.48)	195.53 (38.78)	<0.0001
LDL (mg/dL)	127.39 (34.86)	123.21 (34.02)	128.86 (34.02)	130.09 (36.14)	<0.0001
HDL (mg/dL)	53.90 (14.53)	54.88 (14.57)	54.43 (14.56)	52.39 (14.34)	<0.0001
Triglycerides (mg/dL)	125.11 (78.67)	112.70 (61.69)	121.63 (76.06)	141.00 (92.53)	<0.0001
Fasting glucose (mg/dL)	102.85 (19.44)	101.45 (18.53)	101.77 (18.11)	105.32 (21.29)	<0.0001
HbA1c (%)	5.85 (0.73)	5.86 (0.72)	5.81 (0.69)	5.87 (0.78)	0.378
Creatinine (mg/dL)	0.95 (0.23)	0.95 (0.24)	0.95 (0.19)	0.95 (0.26)	0.964
Albumin (g/dL)	4.64 (0.25)	4.59 (0.24)	4.65 (0.26)	4.68 (0.26)	<0.0001
AST (IU/L)	27.15 (14.93)	24.58 (8.96)	26.02 (11.61)	30.85 (20.80)	<0.0001
ALT (IU/L)	27.94 (17.15)	23.71 (11.47)	26.54 (14.26)	33.56 (22.28)	<0.0001

Values are presented as mean (standard deviation) or percentage.
[a] SMI=ASM/height (m^2).
[b] ≥20g/day.
[c] Moderate physical activity≥5/week or vigorous physical activity≥3/week
ALT: alanine aminotransferase; ASM: appendicular skeletal muscle mass; AST: aspartate aminotransferase; HDL: high density lipoprotein cholesterol; LDL: low density lipoprotein cholesterol; SMI: skeletal muscle mass index.
Serum ferritin tertile (T) levels for men: T1 (<153.5), T2 (≥153.5, <248.0), and T3 (≥248.0).

Supplementary Table 3. Prevalence of multiple adverse body composition according to ferritin group according to cut-off value in women and men.

Number of adverse body composition	Serum ferritin level (ng/ml)			*p* for trend
	Low (<40ng/ml)	Normal (40 to 200 ng/ml)	High (200ng/mL<)	
Women (n = 16,912)	N = 2,032	N = 13,225	N = 1,655	<0.0001
0 (%)	49.1	30.0	22.8	
1 (%)	37.8	45.9	49.3	
2 (%)	12.0	21.9	25.2	
3 (=OSO) (%)	1.1	2.2	2.6	
Men (n = 8,634)	N = 214	N = 4,239	N = 4,181	0.419
0 (%)	29.7	36.4	32.5	
1 (%)	45.8	42.0	46.7	
2 (%)	18.9	18.7	17.7	
3 (= OSO) (%)	5.7	3.0	3.1	

Abbreviations: OSO, osteosarcopenic obesity.

Supplementary Table 4. Multivariate logistic regression analysis for association of ferritin group according to cut-off value with multiple adverse body composition in women and men.

Adverse body composition[a]	Crude	Adjusted OR[b]
	OR (95% CI)	OR (95% CI)
Women (n = 16,912)		
1 (vs 0)		
Low ferritin group	1 (ref.)	1 (ref.)
Normal ferritin group	1.98 (1.79-2.19)	1.48 (1.31-1.68)
High ferritin group	2.80 (2.39-3.26)	1.56 (1.28-1.90)
2 (vs 0)		
Low	1 (ref.)	1 (ref.)
Normal	2.99 (2.57-3.47)	1.95 (1.61-2.37)
High	4.51 (3.70-5.51)	2.01 (1.55-2.61)
3 (=OSO) (vs 0)		
Low	1 (ref.)	1 (ref.)
Normal	3.17 (2.06-4.87)	2.00 (1.16-3.45)
High	4.92 (2.92-8.28)	2.35 (1.22-4.51)
Men (n = 8,634)		
1 (vs 0)		
Low	1 (ref.)	1 (ref.)
Normal	0.75 (0.54-1.03)	0.74 (0.51-1.06)
High	0.93 (0.67-1.29)	0.86 (0.60-1.24)
2 (vs 0)		
Low	1 (ref.)	1 (ref.)
Normal	0.80 (0.53-1.21)	0.87 (0.54-1.38)
High	0.85 (0.57-1.28)	0.95 (0.59-1.51)
3 (=OSO) (vs 0)		
Low	1 (ref.)	1 (ref.)
Normal	0.43 (0.22-0.82)	0.47 (0.22-1.00)
High	0.50 (0.26-0.96)	0.65 (0.30-1.39)

Abbreviations: ALT: alanine aminotransferase; CI: confidence interval; LDL: low-density lipoprotein cholesterol; OR: odds ratio; OSO: osteosarcopenic obesity.
[a]Number of osteoporosis/osteopenia, muscle mass loss, and obesity.
[b]Adjusted for age, hypertension, fasting glucose, heavy drinker, smoking status, LDL, ALT, serum creatinine, and regular physical activity.
Low serum ferritin level: <40ng/ml
Normal serum ferritin level: 40 to 200ng/ml
High serum ferritin level: 200ng/ml<
